# Developing Biomimetic Hydrogels of the Arterial Wall as a Prothrombotic Substrate for In Vitro Human Thrombosis Models

**DOI:** 10.3390/gels9060477

**Published:** 2023-06-10

**Authors:** Jacob Ranjbar, Wanjiku Njoroge, Jonathan M. Gibbins, Paul Roach, Ying Yang, Alan G. S. Harper

**Affiliations:** 1 School of Medicine, Keele University, Keele ST5 5BG, UK; 2 School of Pharmacy & Bioengineering, Keele University, Keele ST5 5BG, UK; 3 Institute for Cardiovascular and Metabolic Research, University of Reading, Reading RG6 6UB, UK; 4 Department of Chemistry, School of Science, Loughborough University, Loughborough LE11 3TU, UK

**Keywords:** animal use alternatives, biomimetic hydrogels, thrombosis, blood coagulation, tissue factor, collagen

## Abstract

Current in vitro thrombosis models utilise simplistic 2D surfaces coated with purified components of the subendothelial matrix. The lack of a realistic humanised model has led to greater study of thrombus formation in in vivo tests in animals. Here we aimed to develop 3D hydrogel-based replicas of the medial and adventitial layers of the human artery to produce a surface that can optimally support thrombus formation under physiological flow conditions. These tissue-engineered medial- (TEML) and adventitial-layer (TEAL) hydrogels were developed by culturing human coronary artery smooth muscle cells and human aortic adventitial fibroblasts within collagen hydrogels, both individually and in co-culture. Platelet aggregation upon these hydrogels was studied using a custom-made parallel flow chamber. When cultured in the presence of ascorbic acid, the medial-layer hydrogels were able to produce sufficient neo-collagen to support effective platelet aggregation under arterial flow conditions. Both TEML and TEAL hydrogels possessed measurable tissue factor activity and could trigger coagulation of platelet-poor plasma in a factor VII-dependent manner. Biomimetic hydrogel replicas of the subendothelial layers of the human artery are effective substrates for a humanised in vitro thrombosis model that could reduce animal experimentation by replacing current in vivo models.

## 1. Introduction

The arterial-wall interaction with blood depends on the integrity of the endothelial lining. In the undamaged artery, the endothelial lining produces anti-platelet and anti-coagulant molecules that act to prevent blood clotting. However, upon damage to the vessel wall, a series of haemostatic reactions are triggered to plug the hole and prevent excessive blood loss. Platelets adhere, activate and aggregate upon the exposed subendothelial matrix sealing the damaged blood vessel (primary haemostasis). Additionally, the localised activation of the blood coagulation system by exposure of tissue factor in the blood vessel wall generates thrombin to amplify primary haemostasis and to generate fibrin to scaffold together with the growing thrombus (secondary haemostasis). Thrombus formation is negatively regulated by paracrine signalling from the endothelium and other blood cells, as well as mechanotransduction of blood flow conditions [1]. Understanding the mechanisms of thrombus formation requires an experimental methodology which best recreates this complex environment.

Currently the best-regarded experimental methodology for understanding the mechanisms underlying in vivo thrombus formation is through real-time monitoring of thrombus formation using intravital microscopy in a variety of arterial thrombosis models. However, the translational potential of these studies may be limited due to species differences in haemodynamics and biochemical environment between humans and mice [2], as well as issues with standardisation of the results. Previous work has suggested that the experimental findings of these arterial thrombosis models are affected by the type and extent of injury induced [3], the vessel bed targeted [4], the age and strain of mice used [5], the anaesthesia and ventilation used [5], as well as research skill in performing the experiments [6,7]. Difficulties in standardising this large array of variables between groups has led to conflicting results even when performing the same injury on the same mouse [8,9]. As different injury models favour different aspects of thrombus formation, it has been suggested that to ensure the validity of findings, results should be drawn across multiple in vivo thrombosis models [3,10,11,12]. An alternative approach would be to develop a physiologically relevant in vitro human thrombosis model to help us better study human thrombus formation outside the body. This could improve pre-clinical predictions of drug performance beyond that currently observed in animal studies [13], as well as the number of mice used in thrombosis research.

Thrombosis-on-a-chip models are being developed to provide a humanised alternative to in vivo animal studies [14,15,16,17]. These in vitro thrombosis models use microfluidic flow chambers coated with thrombogenic substances such as acid-soluble collagen from equine sources and/or recombinant human tissue factor to mimic the procoagulant properties of the subendothelial matrix [18]. These chambers are used to monitor the adhesion, activation and aggregation of fluorescently labelled platelets under physiological flow conditions replicating those found in humans. These models represent the arterial wall as a simplified 2D pro-coagulant surface, which does not contain the anti-thrombotic properties of the endothelial lining or de novo production of collagen and tissue factor by arterial smooth muscle cells. An improved in vitro thrombosis model would fully replicate the pro- and anti-thrombotic properties of the arterial wall.

An effective 3D in vitro model has recently been reported in which endothelial cells are grown on top of a collagen hydrogel containing exogenous lipidated tissue factor [19]. However, this model lacks the presence of subendothelial cells and their effects on the arterial model’s procoagulant properties, which may impact their ability to fully replicate the normal haemostatic responses. Previously we have demonstrated that it is possible to use the principles of vascular tissue engineering to grow an artificial 3D human tissue-engineered arterial construct (TEAC). This was developed from incorporating an intimal layer (comprised of an endothelial monolayer cultured upon a nanofiber mesh) atop a hydrogel replica of the medial layer. The biomimetic hydrogel of the medial layer was created by culturing human coronary artery smooth muscle cells (HCASMCs) in a 3D collagen hydrogel [20]. The combined TEAC construct was found to replicate the haemostatic properties of the native artery. In the intact TEACs, the tissue-engineered intimal layer (TEIL) significantly inhibited human platelet function. However, when the endothelial layer was omitted or damaged, the tissue-engineered medial layer (TEML) hydrogel was found to elicit platelet aggregation—consistent with the properties of the native artery [20,21]. Platelet activation by these TEML hydrogels was found to be dependent on the presence of smooth muscle cells within them [20]. This was correlated with their ability to endogenously produce and secrete their own type 1 and 3 collagen into the surrounding rat-tail collagen hydrogel. As ascorbic acid has been previously shown to increase in vitro production of collagen in 2D culture, experiments were performed to assess if supplementation of the culture media with ascorbic acid could similarly enhance the aggregatory potential of the biomimetic hydrogels through increasing neo-collagen production [22,23].

A realistic 3D prothrombotic substrate for an in vitro humanised thrombosis model also needs to possess measurable tissue factor activity. This will ensure that secondary haemostatic processes can also be effectively triggered upon exposure to human blood samples by the extrinsic pathway of coagulation. Previous studies have demonstrated that smooth muscle cells can express a small amount of tissue factor in human arteries [24,25]. It is possible that the biomimetic TEML hydrogel may also provide an endogenous source of tissue factor to effectively trigger secondary haemostasis for a 3D humanised in vitro thrombosis model. Alternatively, this may be better achieved through incorporating a tissue-engineered adventitial layer (TEAL) to create a 3-layered TEAC, as adventitial fibroblasts are known to be a major source of tissue factor in the arterial wall [24,25]. This can be achieved by creating a biomimetic TEAL hydrogel through culturing human aortic adventitial fibroblasts (HAoAFs) in a 3D collagen hydrogel. In this paper we have developed a fluorogenic assay to assess tissue factor activity in our biomimetic hydrogels, and alongside prothrombin time measurements, used this to assess the activation of the tissue-factor dependent activation of the extrinsic coagulation pathway of our TEML and TEAL hydrogels.

In this paper, we aimed to assess whether biomimetic TEML and TEAL hydrogels alone or in combination could be used to provide an effective 3D prothrombotic substrate for an in vitro humanised thrombosis model. Thus, the production of an effective prothrombotic substrate for thrombus formation created from a 3D replica of the subendothelial tissue layers in this study would provide a key step in developing effective humanised in vitro thrombosis models, thus reducing our reliance on current animal experiments. 

## 2. Results and Discussion

### 2.1. Ascorbic Acid Supplementation Enhances the Rate of Type I Collagen Deposition in Tissue-Engineered Medial Layer Hydrogels

Previously we demonstrated that neo-collagen production by human coronary artery smooth muscle cells (HCASMCs) was responsible for the pro-aggregatory properties of our tissue-engineered medial layer hydrogels. Ascorbic acid is known to be a key regulator of collagen synthesis in vascular smooth muscle cells, both through its role in promoting hydroxylation of key proline residues in the collagen protein, as well as its ability to upregulate collagen gene transcription [22,23]. Supplementing ascorbic acid into the culture media in 2D smooth muscle cell cultures has been shown to enhance collagen production [22], with cell-culture media supplemented with 50 µg/mL of ascorbic acid found to elicit the largest increase in collagen synthesis in primary vascular smooth muscle cells [23]. 

**Figure 1:**
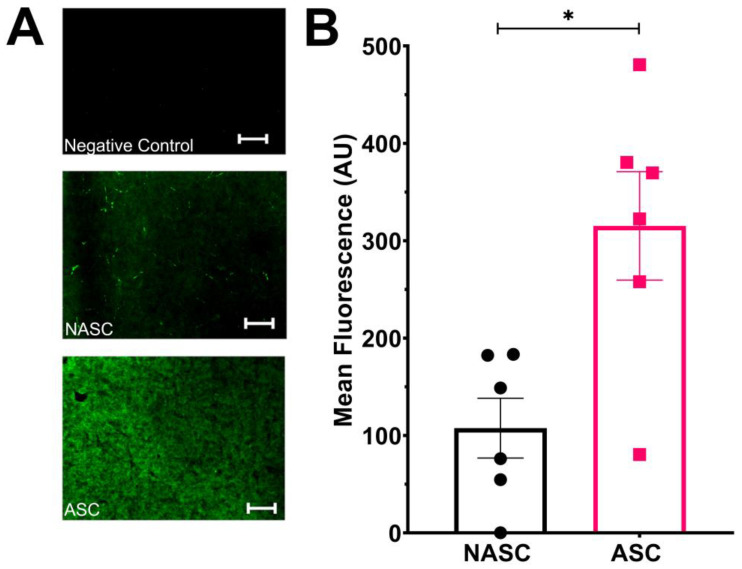
Ascorbic acid supplementation increases the production of neo-collagen by HCASMCs in the tissue-engineered medial layer hydrogels. (**A**) Immunofluorescent imaging of the TEMLs cultured with (ASC) and without (NASC) daily supplementation of 50 µg/mL ascorbic acid. ASC- and NASC-treated TEMLs with mouse anti-human collagen I alpha 1 antibody. Images were taken from the most fluorescent slice within the z-stack. A primary-free negative control is shown in which the TEML was incubated with only the fluorescently labelled secondary antibody (scale bars are 200 µm). (**B**) Mean slice fluorescence of the ASC- and NASC-treated TEMLs. * shows significance of *p* < 0.05 compared to NASC control.

Experiments were performed to assess whether supplementing the HCASMC culture media with 50 µg/mL ascorbic acid enhanced neo-collagen production in the medial layer biomimetic hydrogel compared to samples treated without ascorbic acid supplementation of the culture media. This was assessed using immunofluorescent staining of human type I collagen in TEML hydrogels cultured in either unsupplemented media (NASC) or supplemented with ascorbic acid (ASC), using our previously described method [20]. As the collagen hydrogels are made from soluble rat collagen, we chose a human-specific antibody that will selectively detect neo-collagen produced by the HCASMCs (Figure 1A). Using confocal microscopy, the mean fluorescent intensity through the hydrogels was found to be significantly greater in ASC-treated hydrogels compared to NASC-treated hydrogels (153.5% ± 22.5%; *n* = 5; *p* < 0.05; Figure 1B). Through monitoring cellular metabolic activity using Alamar blue, it was found that this difference was not related to ascorbic acid significantly increasing HCASMC viability or proliferation in the TEML hydrogels (Supplementary materials and supplementary Figure S2 for further details), demonstrating that ascorbic acid enhances neo-collagen production by HCASMCs within the TEML hydrogels.

### 2.2. Increased Neo-Collagen Production Improves the Pro-Aggregatory Capacity of the Medial Layer Hydrogel under Physiological Flow Conditions but Not under Non-Physiological Stirring

**Figure 2:**
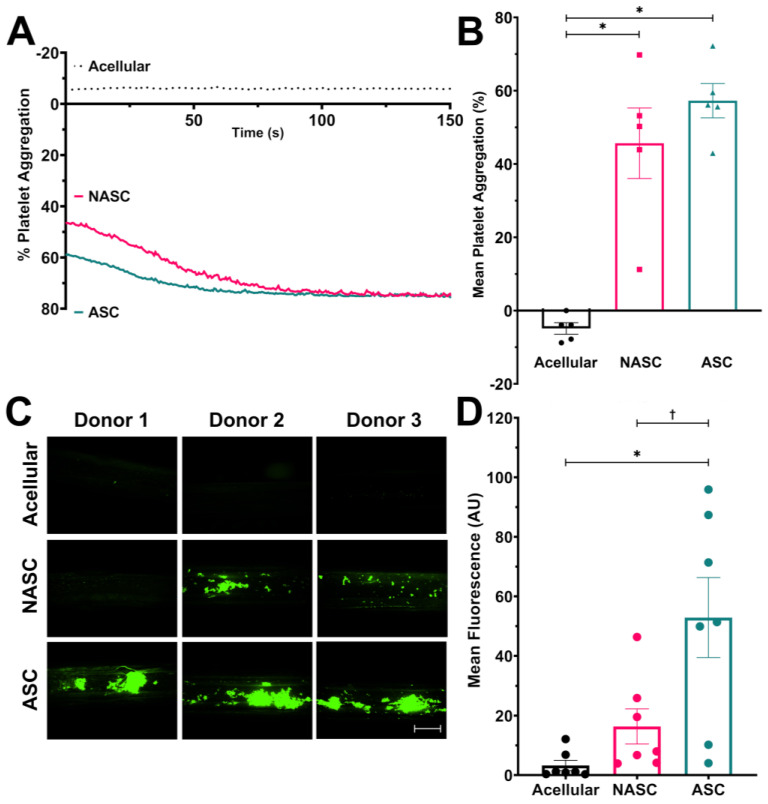
Ascorbic acid supplementation of the TEML hydrogels enhances its pro-aggregatory properties under physiological flow. (**A**,**B**) Washed human platelets were exposed to NASC-treated and ASC-treated TEMLs, and their respective acellular controls, under magnetic stirring for 7 min at 37 °C. Samples were then transferred to a light transmission aggregometer (**A**) A representative aggregometry trace. (**B**) A bar chart summarising the extent of aggregation elicited by the TEML hydrogels and collagen hydrogel following the 7-min incubation period. (**C**) 3D printed chamber utilised to perfuse washed DiOC_6_-labelled human platelets at physiological arterial shear stresses (14 dynes/cm^2^) over the ASC-treated and NASC-treated TEMLs, as well as an acellular collagen hydrogel control. Representative images are shown from samples perfused with blood from three different donors.—Scale bar = 5 mm. (**D**) A summary bar chart showing the mean fluorescent intensity of the TEML surface after platelet perfusion. * indicates *p* < 0.05 compared to acellular control, † indicates *p* < 0.05 compared to NASC.

As collagen is a key agonist for triggering platelet aggregation in vivo [26], experiments were performed to assess whether the increased neo-collagen production elicited by ascorbic acid supplementation enhanced the pro-aggregatory properties of the TEML hydrogels. To assess this, washed human platelet suspensions were exposed to the luminal surface of the ASC- and NASC-treated medial layers, or an acellular collagen hydrogel control under magnetic stirring for 7 min at 37 °C, using a modification of the methodology of Musa et al. [20] (see Supplementary Figure S3). Samples of the platelet suspension were then transferred into a light transmission aggregometer to assess TEML-stimulated platelet aggregation. Washed human platelet suspensions were utilised to prevent any possible contribution from secondary activation via thrombin production. Both NASC- and ASC-treated TEMLs could trigger significant platelet aggregation. However, no aggregatory response was observed in samples exposed to the acellular collagen hydrogel. These results indicate that any platelet aggregatory response was generated by the production of neo-collagen by the HCASMCs and not by stimulation by the collagen hydrogel or acetate sample holder. Although ASC-treated hydrogels tended to trigger greater aggregatory responses at the end of the incubation period than NASC-treated medial hydrogels (45.7% ± 9.6% and 57.3% ± 4.7% for NASC- and ASC-treated hydrogels), the difference was not statistically significant (*p* = 0.30; *n* = 5, Figure 2A). However, the aggregatory response was much less variable between ASC-treated hydrogels. These data suggest that the presence of greater neo-collagen in the ASC-treated medial layer allows the hydrogel to trigger platelet aggregation in a more reproducible manner.

To assess if the enhanced neo-collagen production in the TEML hydrogels could enhance platelet adhesion and aggregation under physiological shear conditions, a 3D-printed flow chamber that could incorporate the medial layer hydrogel was designed (see supplementary Figure S1). This chamber was utilised to perfuse washed DiOC_6_-labelled human platelets at physiological arterial shear stresses (~14 dynes/cm^2^) over the ASC- and NASC-treated TEML hydrogels, as well as an acellular collagen hydrogel control. As can be seen in Figure 2B, the ASC-treated TEML hydrogel could support deposition of extensive platelet aggregates at the end of the 10-min perfusion period, whilst the NASC-treated TEML hydrogel was only able to elicit the formation of small platelet microaggregates. In contrast, the acellular collagen gel allowed for transient adhesion to the substrate but only a minimal number of microaggregates were observed. Image analysis demonstrated that platelet deposition on the surface of the ASC-treated TEML hydrogel (52.9 ± 13.5) was significantly greater than the NASC-treated TEML hydrogel (16.4 ± 5.9; *p* < 0.05, *n* = 7) and the acellular collagen hydrogel (3.4 ± 1.7; *p* < 0.05, *n* = 7, Figure 2C,D). These results demonstrate that enhanced neo-collagen present in the ASC-treated hydrogels is essential for permitting platelet aggregation on TEML hydrogels under physiological shear conditions.

### 2.3. Ascorbic Acid Supplementation Enhances the Procoagulant Properties of the TEML Hydrogels

**Figure 3:**
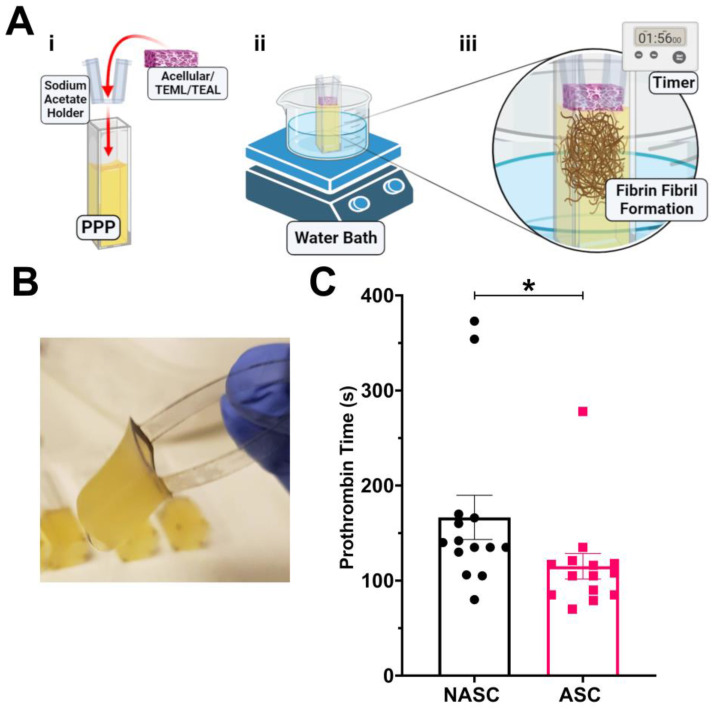
The tissue-engineered medial layer hydrogel can trigger blood coagulation. (**A**) Diagram illustrating the prothrombin time capture for TEML. (i) TEML hydrogels or acellular controls are placed onto sodium acetate frames and placed into contact with the surface of prewarmed, recalcified human platelet-poor plasma (PPP). (ii) The sample is kept warm in a 37 °C water bath. (iii) The time taken between TEML contact with PPP and fibrin formation is recorded. (**B**) A representative photograph of PPP clotting on the surface of the TEML hydrogel post-incubation. (**C**) Bar chart showing the mean prothrombin times recorded for ASC- and NASC-treated TEML hydrogels. * indicates *p* < 0.05 compared to NASC control. Created with BioRender.com.

Upon vascular damage, the exposure of plasma to tissue factor in the arterial wall triggers activation of the blood coagulation cascade. To assess whether the medial layer hydrogels could activate the blood coagulation system, PPP was exposed to either NASC- or ASC-treated TEMLs or acellular hydrogel controls, and the prothrombin time was measured (Figure 3A,B). NASC-treated TEMLs triggered fibrin formation within 165 s ± 23 s after exposure to PPP; however, we found that ascorbic acid supplementation of TEMLs significantly reduced this time to 115 s ± 13 s (*p* < 0.05, *n* = 14, Figure 3C). In contrast to both TEML hydrogels, the collagen hydrogel control showed a markedly longer prothrombin time (400 s ± 52 s; *n* = 10). 

### 2.4. The TEML Hydrogels Trigger the Extrinsic Pathway of the Coagulation Cascade

**Figure 4:**
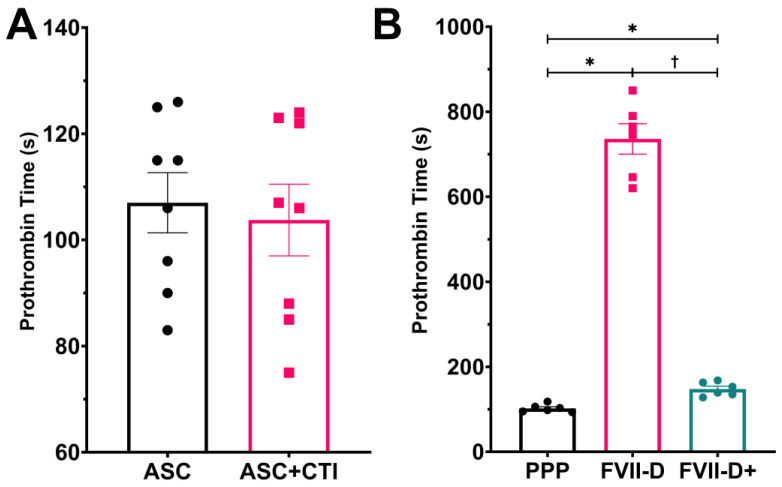
Tissue-engineered medial layer hydrogels trigger blood coagulation through the extrinsic coagulation pathway. (**A**) Prothrombin times were measured for TEML samples which were exposed to prewarmed, recalcified PPP samples pre-treated with either 50 µg/mL Corn Trypsin Inhibitor (CTI) or an equivalent volume of its vehicle, PBS. (**B**) Prothrombin times for TEML hydrogels which were exposed to either PPP from our donor stock, commercially acquired factor VII-depleted plasma (FVII-D), and the same factor VII-depleted plasma which had been exogenously supplemented with of 10 nM inactive factor VII (FVII-D+). * indicates *p* < 0.05 compared to the PPP control. † indicates *p* < 0.05 compared to the FVII-D sample.

Experiments were performed to confirm that the ASC-treated TEML hydrogel was functioning solely through activation of the extrinsic arm of the coagulation cascade. Prothrombin time measurements were performed when the intrinsic pathway was blocked by pre-treating PPP with the factor XII inhibitor, corn trypsin inhibitor (CTI). Prothrombin times elicited by the ASC-treated TEML were not significantly different between those exposed to CTI-treated PPP and those exposed to untreated PPP (107 s ± 5 s and 104 s ± 6 s for PPP treated with and without CTI, respectively; *p* = 0.35, *n* = 8; Figure 4A). 

Prothrombin time measurements were made using factor VII-depleted plasma to confirm whether TEML hydrogels trigger tissue-factor-dependent coagulation of plasma. The TEML hydrogels were exposed to either prewarmed PPP from a healthy volunteer, factor VII-depleted plasma from another donor, and the same factor VII-depleted plasma which had been corrected by addition of exogenous inactive factor VII. The prothrombin time elicited by ASC-treated TEML hydrogels exposed to factor VII-depleted PPP were significantly longer (736 s ± 33 s) than those exposed to untreated PPP (102 s ± 3 s; *p* < 0.05, *n* = 6). Re-addition of exogenous factor VII to the factor VII-depleted plasma counteracted the effect of factor VII depletion on the TEML-triggered prothrombin time (148 s ± 6 s; *p* < 0.05, *n* = 6; Figure 4B). These data demonstrate that the procoagulant activity of the TEML hydrogels is principally mediated through activation of the extrinsic coagulation pathway.

### 2.5. Ascorbic Acid Supplementation Enhances the Tissue Factor Activity of the TEML Hydrogels

**Figure 5:**
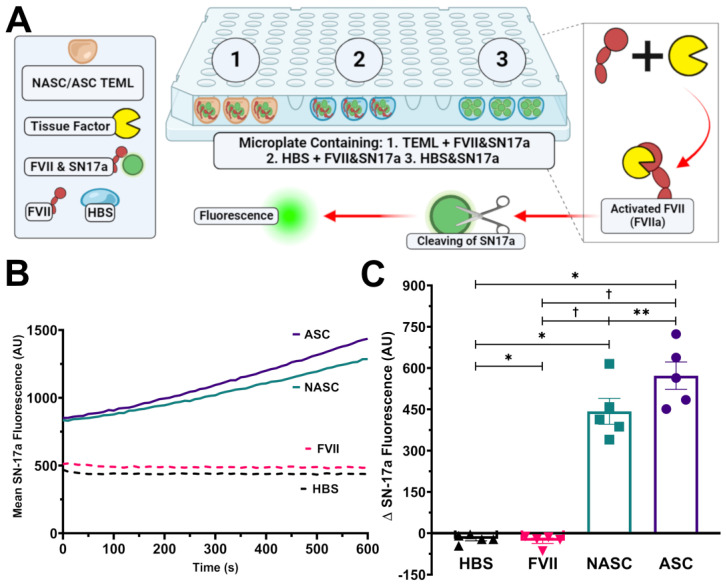
The tissue-engineered medial layer hydrogel possesses measurable tissue factor activity. (**A**) Diagram illustrating a novel method of measuring tissue factor activity in TEMLs cultured on 48-well microplates. Culture media was removed and an HBS solution containing 10 nM inactive factor VII, 1 mM CaCl_2_, and 100 µM SN-17a was added to either TEML hydrogels (1) or an empty well (2; FVII control). Additionally, SN-17a was added to a well of HBS alone (3; HBS control). The well plate was then immediately inserted into the microplate reader and measured fluorometrically. The tissue factor (if any) within the wells will immediately activate the available FVII forming FVIIa. (**B**) Graph showing the mean response observed at all time points across the 5 individual experiments for ASC- and NASC-treated TEMLs as well as experiments in which either the TEML gel (HBS; empty well) or inactive factor VII (FVII) was omitted from the experiment. Note the initial increase in fluorescence in the ASC and NASC samples due to the time delay between adding SN-17a into the wells and the starting of the fluorescent readings. (**C**) A bar chart summarising the change in SN-17a fluorescence observed between the initial reading and that observed after 10 min for TEML samples shown in (**B**). * indicates *p* < 0.05 between this condition and HBS control; † indicates *p* < 0.05 between this condition and FVII control; ** indicates *p* < 0.05 between this condition and NASC sample. Created with BioRender.com.

Blood coagulation can be triggered by activation of either the intrinsic and/or extrinsic pathways [24,27]. Upon arterial damage, the exposure of plasma to tissue factor in the subendothelial space activates the extrinsic coagulation pathways, generating an initial burst of thrombin. A tissue-factor assay was developed to assess whether the medial and adventitial layers contained measurable activity. ASC- and NASC-treated TEAL and TEML hydrogels were cultured in 48-well plates. After culturing, the culture media was replaced with an HBS solution containing inactive factor VII and a fluorescent factor VIIa indicator, SN-17a. SN-17a becomes more fluorescent upon cleavage by factor VIIa, so the rate of increase in fluorescence is dependent upon the tissue factor-based activation of factor VII. This increase in fluorescence was measured using a microplate reader (Figure 5A). 

Control experiments were performed in which the hydrogels were exposed to SN-17a without the addition of inactive factor VII to demonstrate that SN-17a cleavage was dependent upon factor VIIa. Additionally, factor VII- and SN-17a-containing HBS solution were added to empty wells (HBS), to ensure that cleavage was dependent upon tissue factor activity. No fluorescent increase was observed in either of the control experiments, demonstrating that the fluorescent increase was dependent upon tissue factor activation of factor VII by the hydrogels. Both TEML hydrogels triggered rapid increases in fluorescence; however, the rate of increase was significantly faster in ascorbate-treated medial hydrogels (572.1 ± 49.8 and 442.6 ± 47.2 arbitrary unit, respectively; *p* < 0.05, *n* = 5; Figure 5B,C). The initial jump in readings observed in Figure 5A was due to tissue factor-dependent cleavage of SN-17 as the plate enters the microplate reader. These results are consistent with the faster prothrombin time seen in ascorbic-acid-treated TEML hydrogels (Figure 3C). These data demonstrate that ascorbic acid supplementation increases tissue factor activity in TEML hydrogels. 

### 2.6. Ascorbic Acid Supplementation Did Not Enhance the Tissue Factor Activity of Adventitial Fibroblasts

Tissue factor is produced by both smooth muscle cells in the medial layer and fibroblasts in the adventitial layer of the artery [23,24,26,27]. Therefore, experiments were performed to assess whether a 3D tissue-engineered adventitial layer hydrogel could also elicit blood coagulation. To do this a simple 3D tissue-engineered adventitial layer biomimetic hydrogel was made by growing human aortic adventitial fibroblasts in a collagen hydrogel (See Supplementary Materials and Figure S4). Prothrombin time measurements and tissue factor activity assays found that the TEAL possessed a comparable prothrombin time to the TEML and could be observed to have significant tissue factor activity (Figure S5). Interestingly this was not enhanced by the inclusion of ascorbic acid in the culture media—suggesting that the effect of this vitamin on tissue factor activity was specific to HCASMCs.

### 2.7. An Adventitial Layer Is Not Required to Trigger Blood Coagulation

**Figure 6:**
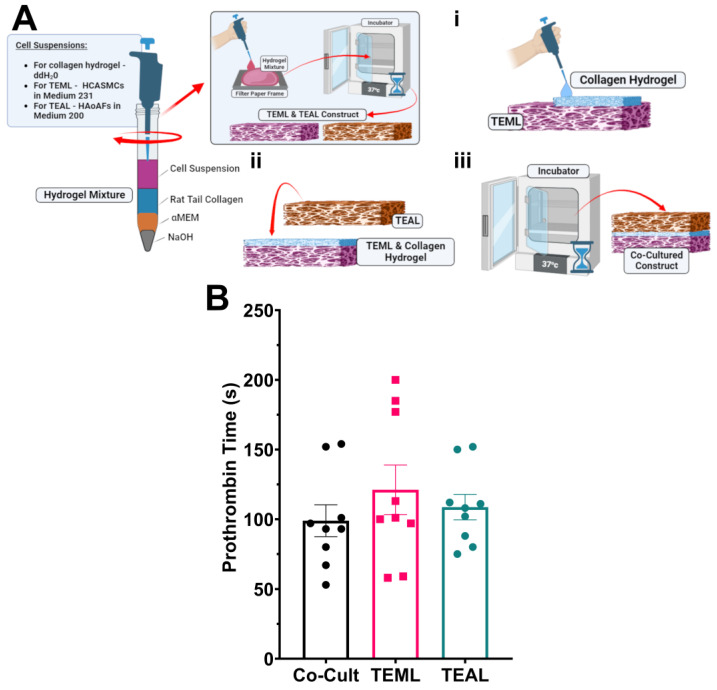
The adventitial layer is not required to effectively trigger blood coagulation in the tissue-engineered blood vessel construct. (**A**) Diagram illustrating the method of TEML-TEAL co-culture fabrication. (**i**) After fabrication of TEML and TEAL hydrogels, rat-tail collagen gel solution was added to the adluminal surface of the TEML hydrogels. (**ii**) TEAL hydrogels were then placed atop the TEML. (**iii**) The hydrogels were then placed into an incubator for an additional 30 min to allow the collagen in between the layers to gelate and bond them together. Co-cultures were then transferred to 24-well plates and cultured in a 65:35 ratio of HCASMC: HAoAF culture media, both supplemented with 50 µg/mL ascorbic acid. (**B**) Mean prothrombin times measure on the TEML layer alone, TEAL layer alone, and TEML-TEAL co-culture. Please note that no significant difference was found between any of the groups. Created with BioRender.com.

Experiments were next performed to assess whether a TEAL-TEML co-culture (Figure 6A) could further enhance the procoagulant activity of the TEML or TEAL hydrogels alone. All hydrogels were cultured in the presence of ascorbic acid throughout the culture period to optimise the coagulation properties of each hydrogel. The prothrombin time measurements for the co-culture were performed with the medial-layer hydrogel in contact with the PPP, to mimic the normal orientation with respect to the bloodstream. No significant difference was found in the prothrombin times of any of these layers (121 ± 18 s, 109 ± 9 s and 99 ± 11 s) for the medial layer alone, adventitial layer alone and adventitial-medial construct, respectively; *p* > 0.05, *n* = 9 for all comparisons; Figure 6B). These results demonstrated that the adventitial layer does not improve the ability of tissue-engineered arterial constructs to activate the blood coagulation system, and as such can be omitted without impairing the ability of tissue-engineered arterial constructs to trigger thrombus formation.

### 2.8. Discussion

In this paper, we have shown that the TEML hydrogels can reliably trigger both platelet aggregation and blood coagulation. These pro-aggregatory and pro-coagulant properties are both enhanced by inclusion of ascorbic acid within the culture media. It is well known that ascorbic acid enhances collagen production through being both an essential cofactor for prolyl hydroxylases and lysyl hydroxylase essential for collagen synthesis, as well as through increasing collagen mRNA stability [22,28,29]. In contrast, we are unable to find any previous data that have shown the effect of ascorbic acid supplementation on smooth muscle cell tissue factor activity. This effect is specific to HCASMCs, as ascorbic acid supplementation did not enhance the tissue factor activity of HAoAFs cultured within a TEAL hydrogel—suggesting that this treatment could regulate HCASMC tissue factor synthesis, cell surface expression or enzymatic activity [30]. The experiments performed in this study are unable to distinguish between these possibilities and further studies will be needed to assess how ascorbic acid treatment of HCASMCs upregulates tissue factor activity in the TEML. Regardless of the mechanisms underlying this effect, ascorbic acid supplementation significantly improves the thrombogenic capacity of the TEML hydrogels through enhancing the endogenous secretion of human collagen and expression of tissue factor activity. The thrombogenic capacity could be further enhanced in future studies by attempting to further optimise the culture conditions to increase the synthesis of neo-collagen and tissue factor within the TEML hydrogels. This could include increasing the starting HCASMC density in the culture, supplementing the culture media with other known stimulators of neo-collagen and tissue factor production (e.g., platelet-derived growth factor [30,31], CD40L [32], C-reactive protein [33,34]), as well as using a bioreactor system to replicate physiological cyclic mechanical stress upon the HCASMCs [35,36,37].

By utilising the synthetic capability of HCASMCs within the TEML hydrogels to produce an endogenous substrate for triggering thrombus formation, we have been able to demonstrate the successful development a 3D prothrombotic substrate that could be used to replace the simplified 2D coatings currently used in vitro human thrombosis models. The TEML hydrogel presented here can successfully trigger platelet activation and blood coagulation by producing endogenous human collagen and tissue factor compared to the use of equine fibrillar collagen and recombinant tissue factor traditionally used in in vitro models [14,15,16,17,18,19]. Thus, using biomimetic hydrogels to develop an artificial human arterial wall can be used to simulate subendothelial matrix synthesis by the cells normally resident in native human arteries. We have previously used scanning electron microscopy, reflectance confocal microscopy and immunofluorescent imaging to demonstrate that HCASMCs within the TEML hydrogel are able to secrete a pericellular matrix containing at least type I and III human collagen, which are the principal collagen components of the vessel wall [20]. In addition, work from other groups has shown that ascorbic acid supplementation of culture media can also increase smooth muscle cell production of type III [23] and type IV collagen [38] as well as fibronectin [39]. This strategy therefore provides the basis for better mimicking the in vivo surfaces that trigger blood clotting. However, a limitation of this study is that we have not fully characterised the composition of the pericellular matrix produced by the HCASMCs. Further studies will be required to characterise the pericellular matrix produced by the HCASMCs and optimise culture conditions to allow it to best reflect the subendothelial matrix of native human arteries.

We have previously reported a method to co-culture an intimal layer with the TEML hydrogel studied here to create a tissue-engineered arterial construct that can replicate the anti-thrombotic properties of the native artery [20,21]. Through incorporating this TEAC within a microfluidic flow chamber to facilitate perfusion under physiological flow conditions with platelet-rich plasma or whole human blood, it will be possible to develop a thrombosis-on-a-chip model that endogenously produces human collagen and tissue factor as its thrombogenic substrate. This differs from other current endothelialised in vitro thrombosis models that either use endothelial activation or devices coating with exogenous sources of these thrombogenic substances [40,41,42]. By replicating current injury models used in in vivo studies (e.g., laser, mechanical or photochemical injury), it will be possible to produce a humanised model in which to perform preclinical screening of anti-thrombotic drugs, whilst also reducing the number of animals utilised in thrombosis research. 

In this paper, a prototype in vitro thrombosis model has been demonstrated using a custom-made 3D-printed flow chamber that can incorporate the TEML. A significant challenge in the use of 3D thrombogenic substrates within in vitro thrombosis models is the difficulty of incorporating these within flow chambers. Whilst the artificial surfaces (e.g., glass, PDMS) of commercially available flow chambers can be easily coated with fibrillar collagen or tissue factor prior to the experiment [14,15,16], a 3D thrombogenic substrate will need to either be set and cultured within the chamber or tightly fitted into a chamber to ensure laminar flow over its surface and prevent blood leakage at the interface of the chamber and 3D substrate. We have used one commercially available flow chamber to facilitate flow at the lower end of arterial shear rates [21], the need to use gaskets to provide a good fit into the chamber is unlikely to support leak-free perfusion at higher shear rates (including those seen in thrombotic environments). Additionally, this chamber requires the production of a much longer gel size than required, which would significantly increase the cost sizes and volume of blood required to conduct experiments. Therefore, to overcome the potential drawback of this methodology, we have utilised 3D printing to produce a custom-sized parallel flow chamber in which to test the pro-aggregatory properties of our TEML hydrogels (Figure S1). This chamber does not provide the benefits of the microfluidic flow channels provided by other in vitro thrombosis models, including reducing the blood volumes required to conduct experiments, use of multiple channels to facilitate high-throughput analysis of a range of experimental conditions, as well as the ability to use imaging data to provide channel structures that replicate the geometries found in vivo [14,15,16,17,40,41,42]. We have subsequently developed a microfluidic chamber which can incorporate either a TEML hydrogel or a complete TEAC to facilitate perfusion with human blood under physiological shear conditions (*Manuscript in preparation*). This take us a step closer to a humanised in vitro thrombosis model that has the potential to endogenously produce all the human prothrombotic factors found in the arterial wall, whilst also recreating the complex physical environments caused by blood flow within the arterial circulation, although there will be much work needed to characterise and validate any such model produced [43].

## 3. Conclusions

In this paper we demonstrate that a biomimetic hydrogel of the medial layer of the native artery can be used to produce an effective 3D prothrombotic substrate that triggers both primary and secondary haemostatic processes. Through future incorporation into a microfluidic flow chamber as part of a complete tissue engineered arterial construct, these hydrogels provide a method to develop improved in vitro human thrombosis models. These would enable us to improve the quality of our pre-clinical testing of anti-thrombotic therapies, whilst reducing the number of animals used in thrombosis and haemostasis research. 

## 4. Materials and Methods

### 4.1. Materials

Human coronary artery smooth muscle cells, Gibco medium 231, Gibco smooth muscle growth supplement (SMGS), α-MEM medium powder, Corning high concentration rat-tail collagen type 1 and Alamar blue cell viability reagent were all obtained from Fisher Scientific (Loughborough, UK). Proliferating Primary Human Aortic Adventitial Fibroblasts, Fibroblast basal medium 2, Fibroblast growth medium 2 Supplement pack, Primary cell detach kit and Live/Dead Staining Kit II were all purchased from VWR International Ltd. (Leicestershire, UK). Human collagen I alpha 1 antibody was purchased from R&D Systems (Minneapolis, USA). Goat anti-mouse IgG (Alexa Fluor 488) secondary antibodies were obtained from Abcam (Cambridge, UK). Trypan blue, paraformaldehyde, and foetal bovine serum (FBS) were supplied by Scientific Laboratory Supplies (Nottingham, UK). SN-17a and FVII-deficient human plasma were purchased from Cambridge Bioscience (Cambridge, UK). Human FVII and corn trypsin inhibitor (CTI) was obtained from Enzyme Research Laboratories (Swansea, UK). Aspirin, Apyrase and luciferin-luciferase were obtained from Sigma Aldrich (Dorset, UK). All other reagents were of standard laboratory grade.

### 4.2. D Culture of Primary HCASMCs and HAoAFs

HCASMCs and HAoAFs were cultured in accordance with the supplier’s recommendations. HCASMCs were cultured in Medium 231 supplemented with SMGS. HAoAFs were cultured in Fibroblast Basal Medium 2 supplemented with the manufacturer’s Fibroblast Supplement pack. The media was changed daily on both cell types until they were 80–90% confluent. HCASMCs and HAoAFs utilised in experiments did not exceed passage 5 and 10, respectively.

### 4.3. Construction of 3D Tissue-Engineered Medial and Adventitial Layers

All reagents were kept prechilled on ice to prevent unwanted gelation during preparation. HCASMCs (for TEML) or HAoAFs (for TEAL) were mixed at a density of 5 × 10^5^ cells/mL into a neutralised solution of 3 mg/mL type I collagen [19] according to the manufacturer’s instructions (Figure S4A). 0.2 mL of the sample was then dispensed directly into 48-well plates, or 1 cm^2^ or 0.5 × 2.2 cm^2^ filter paper frames. The hydrogels were set by incubation for 40 mins at 37 °C, 5% CO_2_. Subsequently, framed hydrogels were transferred into 24-well and 12-well plates, respectively, and cultured with both the smooth muscle cell and fibroblast culture media being supplemented with either 50 µg/mL ascorbic acid (ASC) or unsupplemented culture media (NASC). The media was changed every 24 h. The hydrogels were cultured for 6 days before use in experiments. 

The construction of a 3D tissue-engineered co-culture of the TEML and TEAL was achieved by the addition of 50 µL of neutralised collagen gel solution to the luminal surface of the TEAL hydrogels. TEML was then placed atop the TEAL, and the collagen was allowed to set. The co-cultures were then transferred to 24-well plates and cultured in a 65:35 ratio of HCASMC: HAoAF culture media supplemented with 50µg/mL ascorbic acid. Media was changed every 24 h, and the co-culture was incubated for 6 days before experimentation. 

### 4.4. Preparation of Platelet-Poor Plasma and Washed Human Platelet Samples

This study was approved by the Keele University Research Ethics Committee and conducted in accordance with the Declaration of Helsinki. Blood was donated by healthy volunteers who had given written informed consent. Blood was collected by venepuncture and mixed with either 1 part blood: 9 parts 3.8 % [*w*/*v*] sodium citrate solution for preparation of platelet-poor plasma (PPP), or with 1 blood: 5 acid citrate dextrose solution (ACD; 85 mM sodium citrate, 78 mM citric acid, 111 mM glucose) for preparation of washed platelet suspensions. Subsequently, whole blood was centrifuged at 700× *g* for 8 min to separate whole blood into its constituents. Platelet-rich plasma (PRP) was isolated and 100 µM aspirin and 0.1 U/mL apyrase were added to prevent platelet aggregation. Isolated PRP was centrifuged again at 350× *g* for 20 min to pellet platelets. To prepare PPP, the supernatant was then collected and used for blood coagulation assays. To prepare washed platelet suspensions, the supernatant was removed, and the platelet pellet was then resuspended in HEPES-buffered saline (HBS; 145 mM NaCl, 10 mM HEPES, 5 mM KCl, 1 mM MgSO_4_, pH 7.45), which was supplemented on the day of the experiment with 0.1% [*w*/*v*] bovine serum albumin, 200 μM CaCl_2_ and 0.1 U/mL apyrase (supplemented HBS). Cells were resuspended to give a final cell density of 2 × 10^8^ cells/mL.

For fluorescently labelled washed platelet suspensions, whole blood was collected into ACD containing DiOC_6_ at a final concentration of 1 μM. The blood was mixed with anticoagulant and dye and left to incubate for 10 min at room temperature before centrifugation. The collected PRP was then treated as above for the preparation of washed platelet suspensions.

### 4.5. Immunofluorescent Staining of Human Type I Collagen

TEML cultured for 6 days were washed twice in PBS for 5 min and then fixed for 40 min at room temperature with 4% [*w*/*v*] paraformaldehyde. The fixative was then removed and the TEML hydrogels were washed twice more with PBS for 10 min. The samples were then incubated for 30 min in 0.1% (*v*/*v*) Sudan black B dissolved in 70% ethanol. The hydrogels were then washed twice in PBS with 0.1% [*w*/*v*] Tween 20 (PBST) for 5 min, followed by a final wash for 20 min. The samples were then blocked with 5% (*v*/*v*) goat serum for 30 min at room temperature. This was then removed, and hydrogels were incubated with 1:100 {*v*/*v*} mouse anti-human collagen I alpha 1 antibody diluted in PBST overnight at 4 °C on a rocker. The primary antibody was then removed, and gels were washed 4 times for a duration of 10 min using PBST at room temperature on a rocker. The samples were then incubated overnight at 4 °C on a rocker with Alexa Fluor 488-labelled goat anti-mouse secondary antibody diluted 1:500 in PBST. The antibody was then removed, and the samples washed a further four times for 10 min using PBST at room temperature on a rocker. Collagen staining was assessed by optically sectioning through the sample using the z-stack function of an Olympus FV300 confocal microscope using excitation and emission wavelengths of 473 nm and 490–520 nm, respectively. The mean slice fluorescent intensity was calculated for each sample using ImageJ software. 

### 4.6. Light Transmission Aggregometry 

Aliquots of washed human platelets (1.2 mL) were pipetted into cuvettes and pre-warmed to 37 °C for 10 min under continuous magnetic stirring. Extracellular Ca^2+^ concentration was raised to 1 mM immediately before the experiment. Platelets were then exposed either to ASC-supplemented or NASC-cultured TEMLs, or a cell-free collagen hydrogel, utilising sodium acetate sample holder as previously described by Musa et al. [19]. Following a 7 min incubation at 37 °C with the TEML in cuvettes, the TEML was removed and 450 µL of the washed platelet suspension was transferred into aggregometry tubes containing magnetic stir bars. Changes in light transmission were recorded during constant stirring of the samples at 37 °C. 

### 4.7. Parallel Flow Chamber Experiments

Whole blood was collected into ACD containing a final concentration of 1 μM DiOC_6_. The blood was mixed with anticoagulant and dye and incubated for 10 min at room temperature before centrifugation. The collected PRP was then treated as above for the preparation of washed platelet suspensions. NASC- and ASC-treated TEMLs hydrogels with a dimension of 0.5 × 2.2 cm^2^, and their respective acellular collagen hydrogel controls, were placed into a custom-made 3D-printed perfusion chamber (see supplementary Figure S1). DiOC_6_-labelled washed platelets were perfused over these hydrogels at 14 dynes/cm^2^ for 10 min at room temperature. Platelet aggregation upon the hydrogel was assessed by removing the platelet suspension from the flow chamber and then imaging the surface of the hydrogel with a Nikon ECLIPSE Ti fluorescence microscope using excitation and emission wavelengths of 485 and 501 nm, respectively. ImageJ was used to calculate the mean pixel fluorescence intensity on the surface of the gel.

### 4.8. Prothrombin Assays

PPP was aliquoted into spectrophotometer cuvettes (0.5 mL) and recalcified by the addition of 20 mM CaCl_2_. The PPP was then warmed to 37 °C for 10 min. TEML or TEAL was exposed to the prewarmed PPP on the luminal surface of the hydrogels using the sodium acetate sample holders, as previously described [19]. Prothrombin time was measured as the time taken between hydrogel contact with PPP and the observation of fibrin crystal formation in the PPP sample.

### 4.9. Tissue Factor Activity of TEML and TEAL

TEML and TEAL hydrogels were prepared in 48-well plates for 6 days as described above. The hydrogels were then washed twice for 5 min with HBS at room temperature. Immediately before recording, 400 µL of HBS solution containing 1 mM CaCl_2_, 10 nM inactive factor VII (FVII) and 100 µM SN-17a (the fluorogenic substrate for FVIIa activity, 6-amino-1-naphthalenesulfonamide-based) was added. Cleavage of SN-17a was then measured fluorometrically with a BioTek 2 Synergy microplate reader using excitation wavelengths of 340–380 nm, and emission wavelengths of 518–538 nm. Readings were taken every 10 secs for 10 min at room temperature. 

### 4.10. Statistical Analysis

All values are expressed as the mean ± SEM with the number of observations (n) indicated. Analysis of statistical significance was performed using either a two-tailed Student’s *t*-test or a one-way ANOVA followed by a Post hoc Tukey test. A *p* value of <0.05 was considered statistically significant.

## Data Availability

The data presented in this study are available in the article and supplementary materials provided.

## Supplementary Materials

The following supporting information can be downloaded at: https://www.mdpi.com/article/10.3390/gels9060477/s1, Supplementary methods and results for the following figures: Figure S1: Design and production of perfusion device; Figure S2: Ascorbic acid supplementation of the TEML hydrogels does not impact human smooth muscle cell metabolism; Figure S3: Diagram showing method of light transmission aggregometry experiments modified from Musa et al. [20]; Figure S4: Development of a 3D tissue-engineered adventitial layer (TEAL); Figure S5: The TEAL hydrogel can trigger blood coagulation through its tissue factor activity.
