# Supplementary material for: Developing Biomimetic Hydrogels of the Arterial Wall as a Prothrombotic Substrate for In Vitro Human Thrombosis Models
Source: Gels. 2023 Jun 10;9(6):477. doi: 10.3390/gels9060477 (PMC10298383; doi:10.3390/gels9060477)

**Supplementary Materials**

# Developing Biomimetic Hydrogels of the Arterial Wall as a Prothrombotic Substrate for In Vitro Human Thrombosis Models

**Jacob Ranjbar [1], Wanjiku Njoroge [2], Jonathan M. Gibbins [3], Paul Roach [4], Ying Yang [2] and Alan G. S. Harper [1]**

[1] School of Medicine, Keele University, Keele ST5 5BG, UK
[2] School of Pharmacy & Bioengineering, Keele University, Keele ST5 5BG, UK
[3] Institute for Cardiovascular and Metabolic Research, University of Reading, Reading RG6 6UB, UK
[4] Department of Chemistry, School of Science, Loughborough University, Loughborough LE11 3TU, UK

## 1. Methods

### Alamar blue assessment of HCASMC metabolism

TEMLs were cultured in 48-well plates with their acellular collagen hydrogel controls at a volume of 200 µL. On day 2 of culture, NASC and ASC media were aspirated and fresh NASC- or ASC-supplemented media containing a final concentration of 10% [v/v] Alamar blue. The microplate was then incubated at 37°C, 5% $CO_2$ for 4 hours. After the incubation period, the supernatant of each well was transferred to a new 48-well plate and shielded from light. TEMLs and their respective controls were washed once in sterile PBS before replacing with fresh NASC- or ASC-treated media and placed back into the incubator. The Alamar blue supernatant was then transferred into a microplate reader. Fluorescence from the microplate reader was recorded using 575/15nm excitation and 615/16 emission. This process was carried out on day 2, 4 and 6 of the TEML culture.

### Live/dead cell staining of TEAL hydrogels

Cell viability was assessed using the live-dead cell double staining kit II. Before staining, the medium was removed from TEALs and washed twice for 5 minutes with HBS at room temperature. The live/dead cell staining was conducted according to the manufacturer's instructions. Hydrogels were transferred onto thin coverslips, with HBS added to keep the sample hydrated. The hydrogels were imaged using an Olympus FV300 confocal microscope using excitation/emission wavelengths of 473/519 nm for calcein and 550/578 nm for ethidium homodimer III. Analysis of percentage live/dead cells within the TEAL was carried out using ImageJ by counting cells in each z-stack slice for live cells (green) and then dead cells (red). Live and dead cells were accumulated for all slices and a percentage of living cells were calculated from this.

## 2. Results

### 2.1 Alamar blue assay showed no significant difference in SMC proliferation between ascorbic and non-ascorbic supplemented medial layers

Alamar blue assays were carried out on the medial layers in order to determine whether the contraction of the ascorbic supplemented TEMLs was due to a faster proliferation of the HCASMCs. Alamar blue is a non-toxic, cell permeable fluorescent blue indicator – resazurin is used as redox indicator that undergoes a colour change when reduced by metabolic activity of cells into resofurin. As cellular metabolism is proportional to cell number, this can be used to assess changes in cellular proliferation and viability within the medial layers, and indicate whether the contraction of the ascorbic acid supplemented TEMLs is due to an increased proliferation rate and not another factor.

Alamar blue was added to the TEML media every second day until gel maturation (day 6) and a sample of the media was then read for changes in fluorescence. The results showed a significant difference between acellular collagen hydrogel controls and NASC, ASC TEML hydrogels ($p<0.05$ n=6). However, there is no significant difference in the cellular proliferation between the TEML cultured in the presence and absence of ascorbic acid on day 6 (Figure S2; P=0.15 n=9).

These results therefore showed that the proliferation and viability of HCASMCs within the TEML hydrogels was not significantly affected by ascorbic acid supplementation. These results therefore suggest that increased contraction rate of the TEMLs is more likely due to the migration of smooth muscle cells from synthetic to a contractile phenotype. This demonstrates that the enhance collagen deposition seen in ascorbic supplemented TEMLs is due to an increased production of type I collagen, and is not due to an increased number of HCASMCS within the gel.

### 2.2 Development of the Tissue-Engineered Adventitial layer

As tissue factor is produced by both smooth muscle cells in the medial layer of the artery, as well as fibroblasts in the adventitial layer, it was necessary to assess which the medial layer alone was able to trigger the activation of the extrinsic coagulation cascade, or whether a medial-adventitial co-culture was required. To do this a simple 3D adventitial layer hydrogel was made by growing Human Aortic Adventitial Fibroblasts in a collagen hydrogel. Under light microscopy, HAoAFs were found to have a spindle-like shape with dendrites trying to find its neighbouring cells when cultured both in 2D and 3D environments demonstrating that the HAoAFs maintained their normal morphology in 3D cultures (Figure S4B). Live/dead cell staining of the 3D cultures were assessed for both ASC- and NASC-treated adventitial layer cultures. The HAoAFs remained viable in 3D culture in both the presence (94.2% ± 3.3%) and absence (94.2% ± 1.8%) of ascorbic acid supplementation in the cell culture media, with no significant difference in cell viability discovered between the groups ($P = 0.5$, $n = 5$; Figure S4C). These data demonstrate that HAoAFs are viable in 3D culture and this is not affected by supplementation of ASC into the culture media.

### 2.3 The TEAL hydrogel can trigger blood coagulation through its tissue factor activity.

To assess if the TEAL hydrogel is able to trigger clotting of PPP, prothrombin time measurements were made using both ASC- and NASC-treated TEALs. PPP coagulation was observed using both ASC- and NASC-treated. The Prothrombin time of ASC-treated TEALs (121 ± 16s) were not significantly different when compared to NASC treated TEALs (136 ± 17s; $P > 0.05$ $n = 11$; Figure S5A). Both TEAL hydrogels had a faster prothrombin time than the acellular collagen hydrogel controls (462 ± 39s; $P < 0.05$, $n = 8$). These data suggest that both the medial and adventitial layer hydrogels individually possess the ability to trigger the coagulation cascade – however ascorbic acid only enhances the pro-coagulant activity of the TEML.

Further experiments using the SN-17a tissue factor assay, found that both ASC- and NASC-treated TEAL hydrogels possessed measurable tissue factor activity. However, there was no significant difference observed in ASC-treated TEAL hydrogels (409.8 ± 12) compared to NASC– treated TEAL hydrogels (375.7 ± 9.4; $P = 0.37$, $n = 5$; Figure S5 B,C). These results are consistent with the prothrombin time measurements recorded from the ASC- and NASC-treated TEAL hydrogels. These data confirm that the ascorbic acid enhancement of tissue factor activity is specific to TEML hydrogels. The differential effect of ascorbic acid supplementation on the prothrombin time on the medial and adventitial layer hydrogels suggest that this effect is due to a specific effect on HCASMCs.

## 3. Supplementary Figure Legends

**Figure S1. Design and production of perfusion device.** (A) Autodesk Fusion 360 render of perfusion channel from the perspective of the inlet channel (B) Table showing methods of perfusion channel production and flow properties. (C) Diagram showing a top down cross section of the perfusion device. (D) Diagram showing the cross section of the side of the device. (E) Diagram showing the bottom up cross section of the perfusion device. (F) Diagram showing the cross section of the inlet channel of the perfusion device, highlighting the buffer zone. The buffer zones utilise slopes to gradually expand the inlet/outlet channels. This reduces turbulent flow and allows the TEML within the perfusion channel to experience mainly laminar flow.

**Figure S2. Ascorbic acid supplementation of the TEML hydrogels does not impact human smooth muscle cell metabolism.** Alamar blue is a resazurin based solution that is used to determine cell health by using the living cells abilities to reduce resazurin to resorufin. Alamar blue was therefore used as a method in determining smooth muscle cell proliferation and/or viability within the TEML hydrogels. Alamar blue was used in accordance with manufactures instructions. Alamar blue was added on day 6 of the culture period of ASC- and NASC- treated TEMLs cultured on 48-well plates. As shown in Figure S2, there was no significant difference in Alamar Blue measurements of ASC- (16269 ± 1185) and NASC- (14872 ± 1212) treated hydrogels at the end of the 6-day culture period ($P = 0.15$, $n = 9$).

**Figure S3. Diagram showing method of light transmission aggregometry experiments modified from Musa *et al*.** (A) NASC- or ASC- treated TEMLs, or their respective acellular controls were placed into sodium acetate frames and lowered into a cuvette containing $ca^{2+}$ activated washed platelet solution (WPS). (B) Cuvettes were then placed in a water bath under magnetic stirring for 7 mins at 37$^{o}$C. (C) Sodium acetate frames containing NASC- or ASC- treated TEMLs, or their respective acellular controls were removed from the cuvette and 450 µL WPS was then transferred into aggregometer tubes and placed into a light transmission aggregometer to measure platelet activation.

**Figure S4 - Development of a 3D tissue-engineered adventitial layer (TEAL).** (A) Diagram illustrating the novel method of TEAL fabrication. (i) HAoAFs were mixed into a neutralized solution of rat tail type I collagen made according to the manufacturer's instructions. (ii) The cell-collagen mixture was then pipetted into either 48-well plates or 1cm$^2$ or 0.5x2.2cm$^2$ filter paper frames. (iii) The hydrogels were set by incubating them for 40 minutes at 37°C, 5% $CO_2$. (B) Light microscopy of HAoAFs in 2D culture. HAoAFs form the expected spindle-like shapes, stretching out to neighbouring cells. (C) Confocal image of a live/deal dead cell stained TEAL. Please note the HAoAFs show similar spindle-like morphology to that observed in 2D culture. (D) Percentage of living HAoAFs within ASC- and NASC-treated TEAL hydrogels.

**Figure S5. The TEAL hydrogel can trigger blood coagulation through its tissue factor activity**. (A) Bar chart showing the mean prothrombin times recorded for ASC- and NASC-treated TEAL hydrogels (B). Graph showing the mean response observed at all time points across the 5 individual experiments for ASC- and NASC-treated TEALs, as well as experiments in which either the TEAL gel (HBS; empty well) or inactive Factor VII (FVII) was omitted from the experiment. (C) A bar chart summarising the change in SN-17a fluorescence observed between the initial reading and that observed after 10 mins for TEAL samples. - * indicates $p < 0.05$ between this condition and HBS control. †indicates $p < 0.05$ between this condition and FVII control.

**Supplementary Figure S1**

A

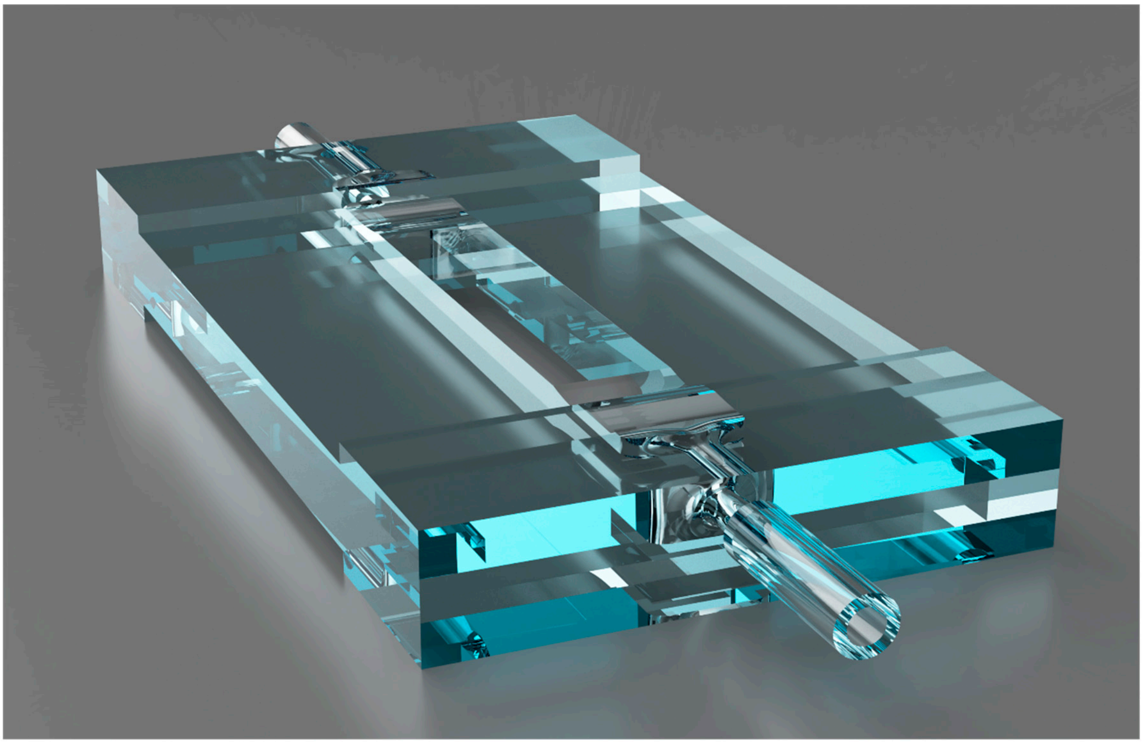

B

| | |
|---|---|
| **Design Software** | AutoDesk: Fusion 360 |
| **Slicing Software** | Chitubox 64 |
| **Printer Used** | Elegoo Mars LE |
| **Z Resolution (layer height)** | 0.025mm |
| **X/Y Resolution** | 0.047mm |
| **Material Used** | Elegoo Transparent/Grey Resin |
| | |
| **Post Processing** | Isopropyl Wash (5-10min),<br>UV Curing (10-20 mins with rotation) |
| | |
| **Total Length** | 61mm |
| **Total Width** | 25mm |
| **Total Depth** | 5.4mm |
| | |
| **Chamber Length** | 25mm |
| **Chamber Width** | 5mm |
| **Chamber Depth (excluding TEML)** | 3mm |
| | |
| **Flow Rate** | 0.07cm$^2$/s |
| **Shear Stress through chamber** | ~14dyne/cm$^2$ |
| **Viscosity value used** | 1.5Cp |

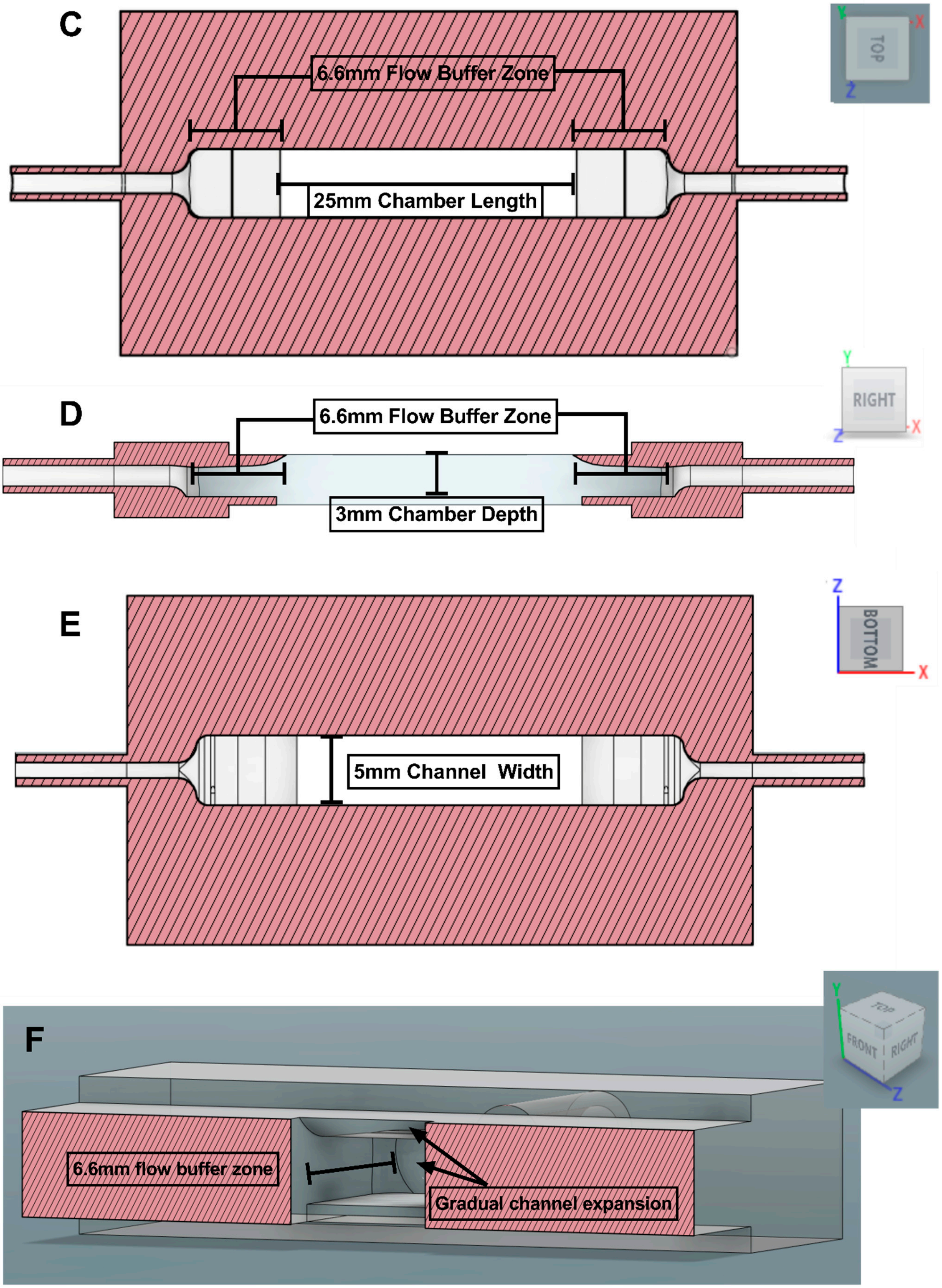
C
6.6mm Flow Buffer Zone
25mm Chamber Length
TOP
D
6.6mm Flow Buffer Zone
3mm Chamber Depth
RIGHT
E
5mm Channel Width
BOTTOM
F
6.6mm flow buffer zone
Gradual channel expansion
TOP
FRONT
RIGHT

**Supplementary Figure S2**

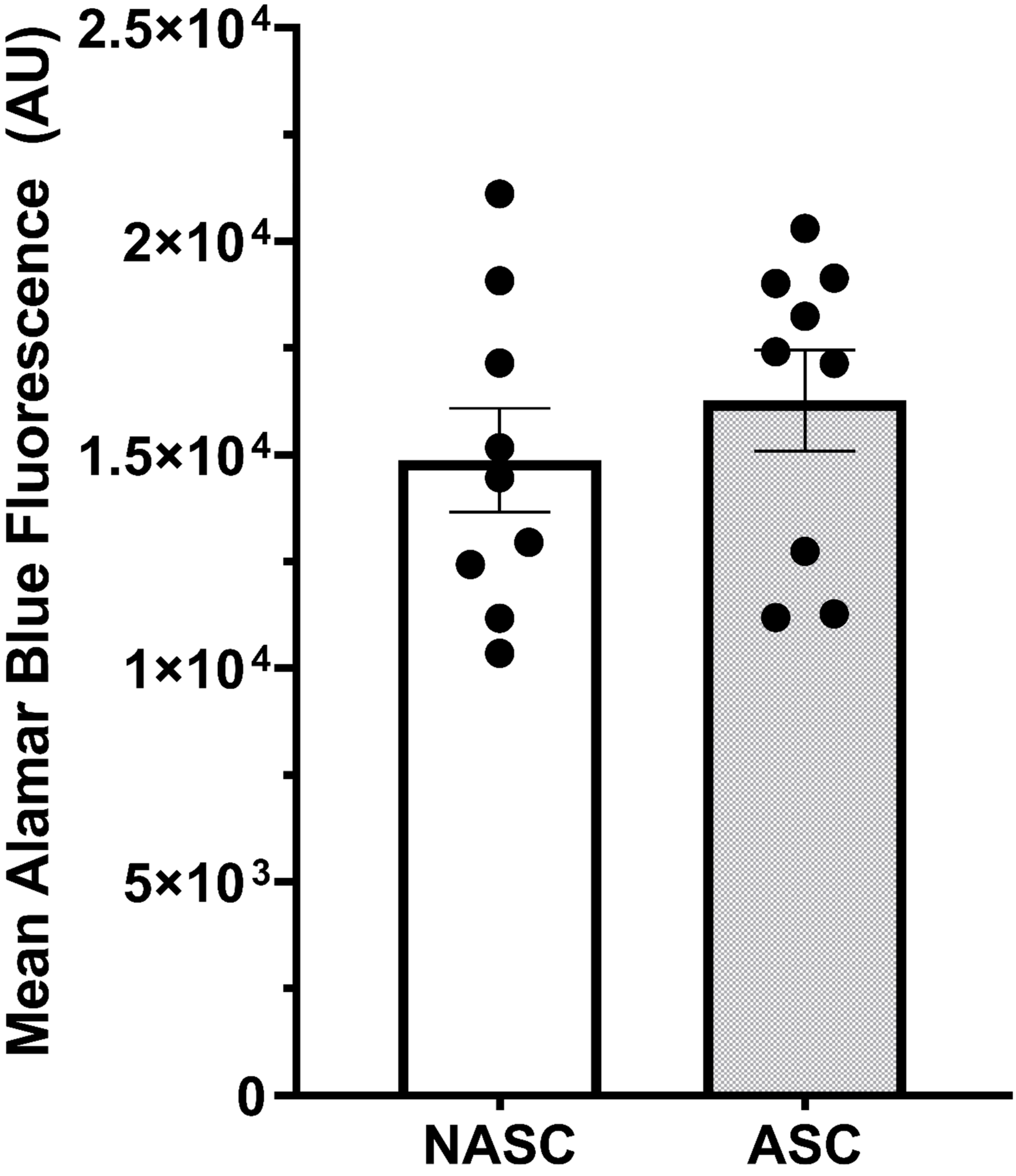

## Supplementary Figure S3

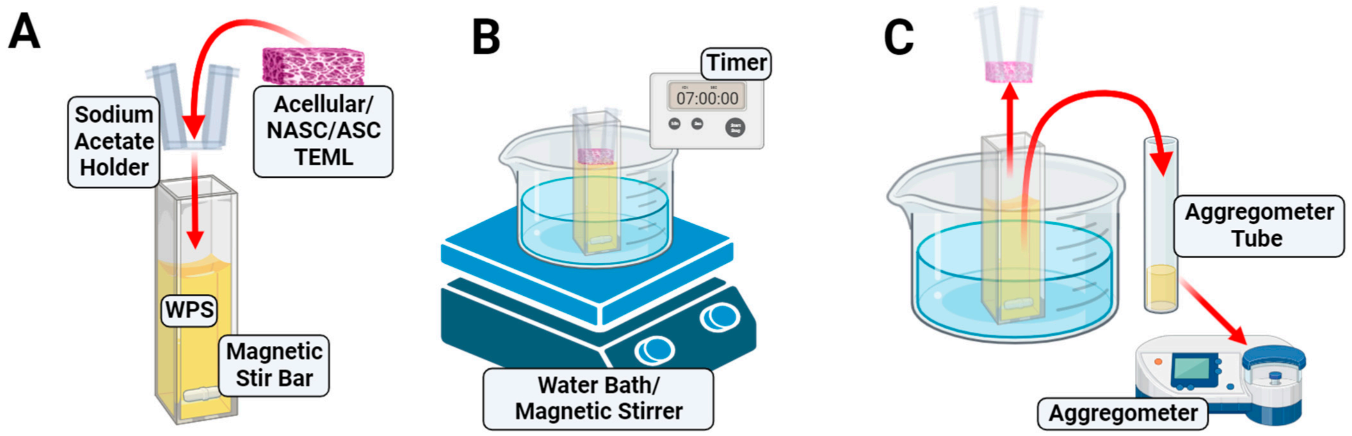

## Supplementary Figure S4

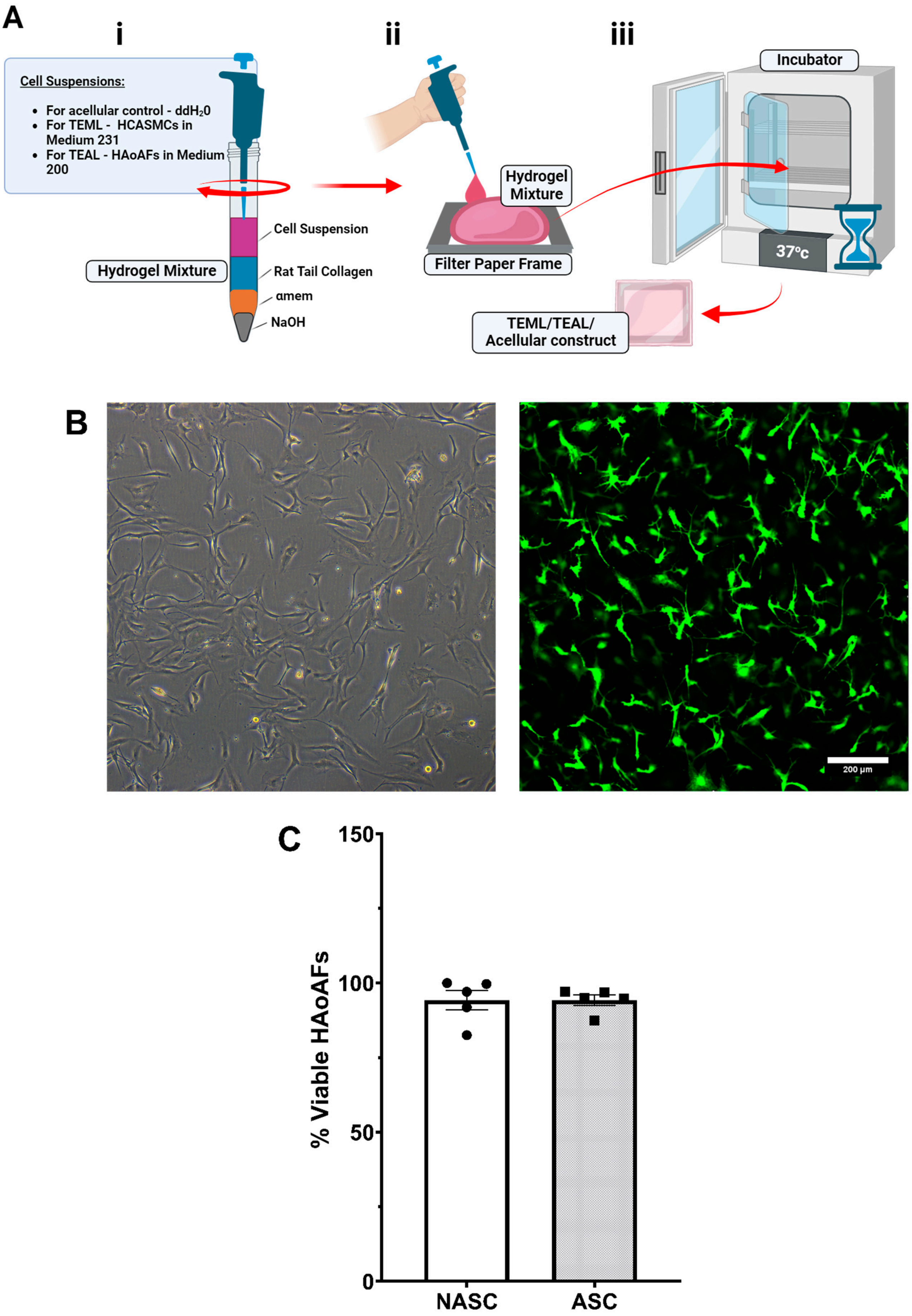

**Supplementary Figure S5**

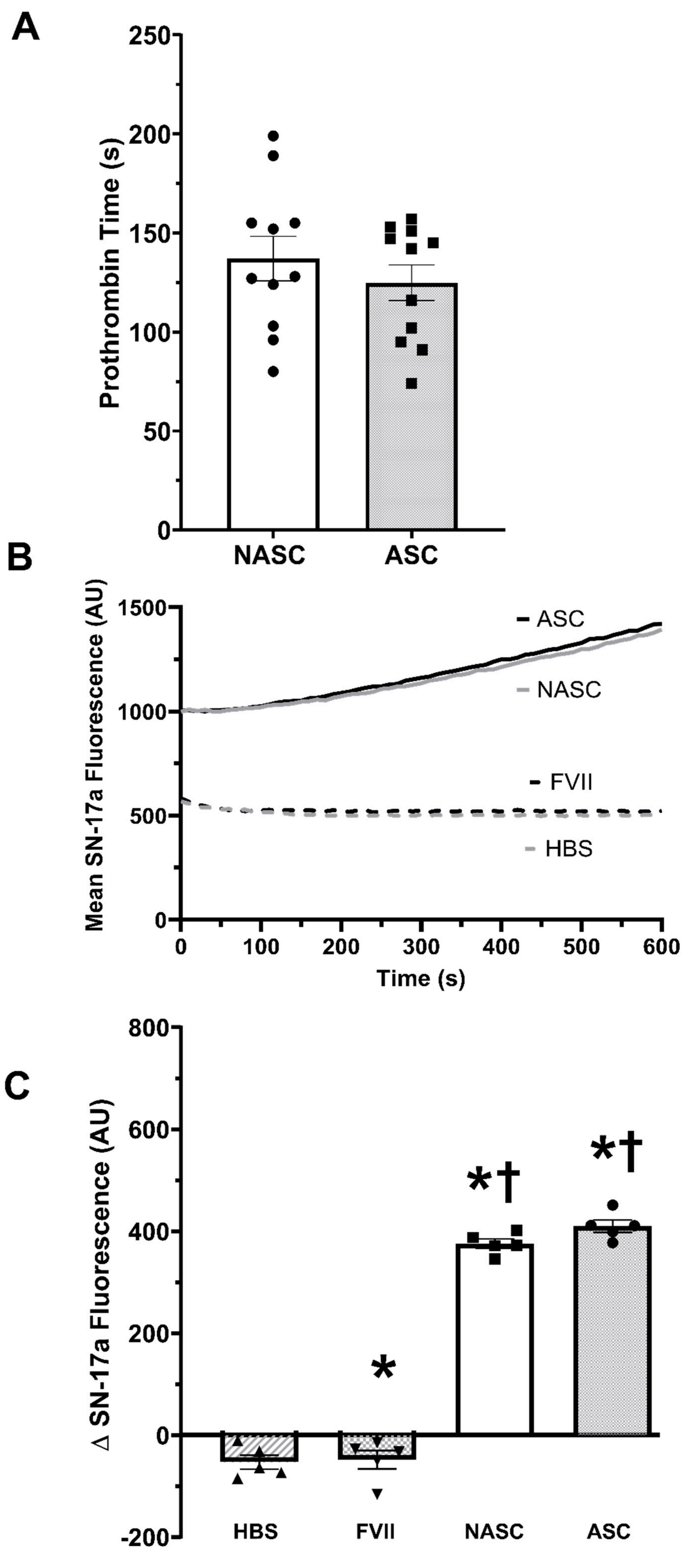

Supplement: Supplementary file 1 [file gels-09-00477-s001.zip › gels-2417999-supplementary.pdf]
